# Crito, in Reply to a Derbyshire Practitioner

**Published:** 1808-03

**Authors:** 


### *To the Editors of the Medical and Physical Journal.*

GENTLEMEN,

IN my last Reply to the Derbyshire Practitioner, (in your No. 104.) I have expressed my opinion of compression, and where it may be useful; at the same time I have shown, that in cases of diffused aneurism it is certainly prejuidcial. In the case I recorded, the hæmorrhage was at first stopped by compression, to which I attributed the great swelling, and, notwithstanding the great confidence of the Derbyshire Practitioner, would have alarmed him a little; compression in a case of this kind could never be expected to stop the hæmorrhage. He is not satisfied with my account of the tibial arteries; an anatomist, who can dissect the brain with such great nicety, whose feelings are so much pained by a wrong push of the scalpel on a dead subject, is not easily satisfied.

This particular observer is not, however, entirely pleased with Dr. Cayley's case, when he says, "*We* have already observed on this head, that it would have added greatly to the interest of Dr. Cayley's case, if he had given us the precise situation of the wound; we could then have spoken more determinately about, not only that, for it would besides have been very sastisfactory to have known what what part of the artery it was, that Dr. Cayley was so fortunate as to obliterate by compression. It being a fact, of which we all must be apprised, that those points of the arteries that are in a manner beyond the reach of operation, would, from being so surrounded and cushioned as it were with flesh, be the most difficult to obliterate by compression." Is it not evident from this paragraph, that he must also doubt whether it was the anterior tibial artery that was cut. I did not know that he could distinguish the anterior tibial artery by the " first gush of blood," or " the instrument that gave the wound." An artery may be wounded by any sharp instrument; it is therefore absurd in him to say, that he could know the artery from this circumstance. If he had said we could know from the exact situation of the wound, or aneurism, or the colour and flow of blood per saltum, it would have been more to the purpose. But, in Dr. Cayley's case we are not blessed with such information ; and as for the hæmorrhage, it was stopped before Mr. Strother's arrival. He observes, that the tumour could afford us the necessary information, and yet he appears to be dubious of it, when he says, " that those points of the arteries that are in a manner beyond the reach of operation, would, from being so surrounded and cushioned as it were with flesh, be the most difficult to obliterate by compression." He remarks, that Dr. Cayley observed, " that the pulsation was not only tangible but visible across the room." Surely, this had not been so tangible or visible to Mr. Strother, or he would not have poulticed the tumour for such a long time.

He next recurs to my case of wounded ulnar artery. There is an invincibility in truth, of which I will never be ashamed, therefore, I confess that I was not able to take up the artery (which was fairly cut across and retracted within a mass of corruption), with the tenaculum—but it was to appearance properly secured with the needle : — This, however, gave way from the corrupted state of the wound, and another attempt was made with very little prospect of success. Had the artery been secured by the tenaculum during this corrupted state of the wound, it must have been equally as unsuccessful as the needle. The only time for properly securing the artery was immediately when the wound was received, and not two days after; I therefore much doubt whether the Derbyshire Practitioner, with all his self-confidence, could have treated the *perverse creature,* as he is pleased to term it, in a better manner.

manner. The perverse creature is not at all times so easily hooked as the Derbyshire Practitioner would insinuate, Anatomical connoisseurs must admire the fruitful genius of this great anatomist, who is as little pleased with the old nomenclature as the public dissection at Derby.—He says, " it is self-evident that I did not get round the artery, from the frequent and quick returns of hæmorrhage, as well as from the man's requiring at a future time, a certificate to exempt him from military duty.

To this I reply, that it frequently happens after amputation of any of the extremities, where we have the most favourable opportunities of securing the arteries, that a hæmorrhage will return from the suppuration loosening the ligatures too quickly; but, in a case where there was not the possibility of securing the artery for corruption, attended with great swelling and tension of the parts; is it so surprising to meet with a recurrence of hæmorrhage, especially when the swelling continuing to increase, tended much to weaken the power of the ligature?

To the second evidnce of my not getting round the artery, I have only to observe, that the man is at present serving his Majesty in the Royal Artillery. The inspecting surgeon must have seen that he had the perfect ability of his hand previous to his passing him. He continues to say, " Whereas, if he had succeeded in getting round the artery in this way, that is, by a determined stroke of the needle, he must unavoidably have encompassed the nerve, and in that case, the *contracted* fingers of the sufferer would have exhibited a sad and unequivocal testimony of his inability.

Now, Gentlemen, had I been so unfortunate as to have included the nerve, whether would a *contracted* state of the fingers, or a loss of feeling been the consequence? I think he is as unfortunate in his application of the word *contraction* here, as Dr. Cayley was in his term, cicatrization.

Had the tendons of the wrist been cut, I believe the hand would have suffered contraction; and if the principal nerves, a loss of feeling. But might not the nerve have been cut when he first received the wound? Had it been so, and the Derbyshire Practitioner examined the man's hand, and discovered a loss of feeling, he would have pronounced it contraction, and that I had occasioned it by including the nerve in the ligature. Query, As the fingers are also supplied with branches from the radialis, would a loss of feeling have been the consequence had the ulnaris been cut? With

With respect to the case that terminated fatally, I can only say, that I did not attend the patient, nor witness the state of the wound. I was far from considering it a subject of pleasantry, yet when I observed the Derbyshire Practitioner ordering wounded arteries to be obliterated by a single pull, I could not help noting this remarkable expression. Altho' the Derbyshire Practitioner tells us he is of no professional consequence himself, yet from his writings does he wish to impress us with this belief, when he assures Crito, that it would be a matter of painful consideration with him, to have a case of wounded ulnar artery simply terminate fatally. Yes, I acknowledge, that this to appearance does not carry terror with it; but how often do we find simple cases, contrary to our expectation, terminate fatally. Whatever may be the Derbyshire Practitioner's professional consequence, this much I can say, that if extensive practice, frequent opportunities of performing all the different operations of surgery (including every kind of amputation and lithotomy) can give professional consequence, the gentlemen who attended that case were entitled to the term. I will not, however, take upon me to say, that having these opportunities will give them a superior genius to other men, or enable them to succeed in a case of this kind better than the Derbyshire Practitioner or myself. I wish it has not the very opposite effect, by making them pay too little regard to it, not considering the fatal consequence that neglect or oversight frequently occasions.

CRITO.

*December*, 1807.

